# Improved dynamics of sharing research findings in the COVID-19 epidemic compared with the SARS and Ebola epidemics

**DOI:** 10.1186/s12889-020-10116-6

**Published:** 2021-01-09

**Authors:** Javad Khanali, Mohammad-Reza Malekpour, Ali-Asghar Kolahi

**Affiliations:** Social Determinants of Health Research Center, Shahid Beheshti University of Medical Sciences, Tehran, Iran

**Keywords:** COVID-19, SARS, Ebola, PubMed database, Data sharing, Policymaking, Research topics

## Abstract

**Background:**

When a new or re-emergent pathogen, such as SARS-CoV-2, causes a major outbreak, rapid access to pertinent research findings is crucial for planning strategies and decision making. We researched whether the speed of sharing research results in the COVID-19 epidemic was higher than the SARS and Ebola epidemics. We also researched whether there is any difference in the most frequent topics investigated before and after the COVID-19, SARS, and Ebola epidemics started.

**Methods:**

We used PubMed database search tools to determine the time-period it took for the number of articles to rise after the epidemics started and the most frequent topics assigned to the articles.

**Results:**

The main results were, first, the rise in the number of articles occurred 6 weeks after the COVID-19 epidemic started whereas, this rise occurred 4 months after the SARS and 7 months after the Ebola epidemics started. Second, etiology, statistics & numerical data, and epidemiology were the three most frequent topics investigated in the COVID-19 epidemic. However, etiology, microbiology, and genetics in the SARS epidemic, and statistics & numerical data, epidemiology, and prevention & control in the Ebola epidemic were more frequently studied compared with other topics. Third, some topics were studied more frequently after the epidemics started.

**Conclusions:**

The speed of sharing results in the COVID-19 epidemic was much higher than the SARS and Ebola epidemics, and that there is a difference in the most frequent articles’ topics investigated in these three epidemics. Due to the value of time in controlling epidemics spread, the study highlights the necessity of defining more solutions for rapidly providing pertinent research findings in fighting against the next public health emergency.

## Background

In December 2019, several cases of pneumonia of unknown cause were reported in Wuhan, China [1]. On 7th January 2020, a novel coronavirus, now named SARS-CoV-2, was identified as the responsible infectious agent of the coronavirus disease-2019 (COVID-19) epidemic [1, 2]. Due to the quick spread, the COVID-19 became pandemic [3], and unfortunately, over 32.7 million cases and 991,000 deaths were reported globally until the 27th September [4].

When a new or re-emergent pathogen, such as SARS-CoV-2, causes a major outbreak, rapid access to pertinent research findings is crucial for planning control strategies, and decision making [5, 6]. Former experiences of battling against the SARS and Ebola epidemics proved that shortcomings in sharing data and research findings could lead to unnecessary suffering and death and disastrous public health consequences [5]. Both the SARS and Ebola experiences were accompanied by late official reports of the epidemics, comparatively slow dissemination of surveillance data, unclear criteria for data sharing, and the unwillingness of several individuals and organizations to share vital data in real-time [6–9]. Sensing these failures in data sharing policies led to the agreement on the need for timely and transparent sharing of data and results, especially in public health emergencies, in September 2015 [5, 10, 11]. Furthermore, the International Committee of Medical Journal Editors stated that pre-publication information that is critical for public health is to be shared with WHO– a commitment echoed by several leading journals in the context of the COVID-19 response [11].

Besides the rapidity of sharing articles, the topics covered by the articles published after the start of an epidemic are important too. The articles could cover various aspects of the disease like diagnosis, treatment, epidemiology, transmission, and so [12], leading to answering different research questions important for policy setting and controlling the epidemic [13, 14]. Therefore, classifying the articles published in the epidemics period based on the topics covered in them could represent research questions that were essential to answer in epidemics. Hence, these topics should be focused on and financially supported in fighting against the next disease outbreaks.

However, to our knowledge, the dynamics of sharing research results regarding the COVID-19 epidemic has never been compared with the previous epidemics like SARS and Ebola to allow proper judgment about the effectiveness of the new data-sharing policies employed in the COVID-19 epidemic. Besides, there is a scarcity of studies classifying the outbreaks-related published documents based on their topics to determine the necessary research topics in fighting against the epidemics. In this study, we asked whether the speed of sharing research results in the COVID-19 epidemic was higher than the SARS and Ebola epidemics. We also asked which topics were more investigated in the COVID-19, SARS, and Ebola epidemics. To answer these questions, we retrospectively analyzed the PubMed documents pertaining to every three mentioned epidemics.

## Methods

### Study design

To determine the speed of sharing research data and results in the COVID-19 epidemic, we measured the time-lag between the epidemic start time and the rise in the number of coronavirus-related documents indexed in the PubMed database. The PubMed database enables easy access to the Create Date (CRDT) of each article, which is the date that the article was added to the database [15]. This date is important because the articles become searchable for the research community since then. We determined the number of PubMed publications pertaining to coronaviruses indexed in each week by finding articles that have the term “coronavirus” in their “title/abstract” and indexed in “Date-Create (CRDT)” of the week range. Forty-eight weeks before the epidemic start-time was set as the control period (from 6th January to 8th December) [1, 16], and the number of documents per week for this period was assumed as the baseline level. Afterward, the number of documents per week after the epidemic start-time was analyzed, and the week in which the numbers of documents increased significantly compared to the baseline level was determined. Then, the time range between this week and the epidemic start-time and declare-time was calculated. We also did the same for the SARS and 2014 Ebola epidemic in West Africa by changing the time scale to month. Therefore, forty-eight months before each epidemic start-time was considered as the control (1st November 1998 to 1st November 2002 for the SARS [17], and 1st December 2009 to 1st December 2013 for the Ebola epidemic [18]). The time frame change was made because of the low number of publications per some weeks for these two epidemics (even zero for some records). In the case of the Ebola epidemic, the term “Ebola” was searched in the “title/abstract.”

To classify the topics investigated in the articles before and during the COVID-19, SARS, and Ebola epidemics, we used the PubMed Medical Subheading (MeSH) database. MeSH terms and subheadings are controlled vocabularies for indexing and searching biomedical literature, which is used as an indicator for the topic of an article [19]. Eleven Mesh subheadings were chosen to classify the articles’ topics into eleven categories: diagnosis, drug therapy, epidemiology, etiology, genetics, immunology, microbiology, prevention and control, statistics & numerical data, transmission, and organization & administration. Each category’s exact definition is presented in the PubMed MeSH database in the subheadings part [20]. For the COVID-19, the year 2019 was considered before the epidemic period, and the year 2020, up to 10th April, was considered the epidemic course. For the SARS and Ebola epidemics, the years 2002 and 2013 were considered as before the epidemics period, respectively and, the years 2003 and 2014 were considered the epidemic course. This consideration’s logic was based on the previous searches, which showed a marked rise in the number of relevant articles in 2003, 2014, and 2020.

To find the number of articles having each topic in them, we found the number of PubMed publications that indexed in the desired “Date-Create” and have the term “coronavirus” (“Ebola” in the case of the Ebola epidemic) in their “title/abstract” and assigned to the desired MeSH subheading. Samples from the total search results were screened manually to assess the validity of the search method. After approving the search method’s validity, all of the results were included in the analysis, and no exclusion was performed. A similar process was done for finding the records pertaining to the SARS and Ebola epidemics. Notably, each article could be assigned to a few of these subheadings or none of them; therefore, the sum of the subheadings’ frequency was not equal to the total number of articles indexed.

Finally, to determine the topics that were more investigated during the epidemic courses, two measures were compared between before and during each epidemic: First, the rank of each topic in frequency, and second, the proportion of each topic frequency to the total number of search results (relative frequency).

### Statistical analysis

For analyzing the rise in the number of publications after each epidemic emerged, we omitted outlier records in the control period by considering any value that lies more than one and a half times the interquartile range (IQR) beyond the first and the third quartiles. Therefore, two records were omitted from the SARS and Ebola control periods (October 2011 and December 2012 in the Ebola and December 1998 and January 2002 in the SARS epidemic control period). Then, the Shapiro-Wilks test was performed to confirm that all of the control periods follow a normal distribution [21]. The records belong to after each epidemic start-time was analyzed using a one-sample z-test to determine the first record that shows a significant increase compared to the control period. All Data are summarized as mean (SD), and we considered differences at *p*-value < 0.01. Data analysis was conducted using Python (version 3.6). The SciPy library (version 1.4.1). Data visualization was performed using Tableau Desktop (version 2020).

For comparing the proportion of each topic frequency to the total number of search results (relative frequency of topics) between before and after each epidemic start-time, the Z test for two population proportions was performed. Using the analysis, we determined the topics that their relative frequency was increased significantly after the epidemics start-time. We considered increase at *p*-value < 0.01. The data was visualized in the figures using Word document (version 2016).

## Results

### The COVID-19 epidemic

**Fig. 1:**
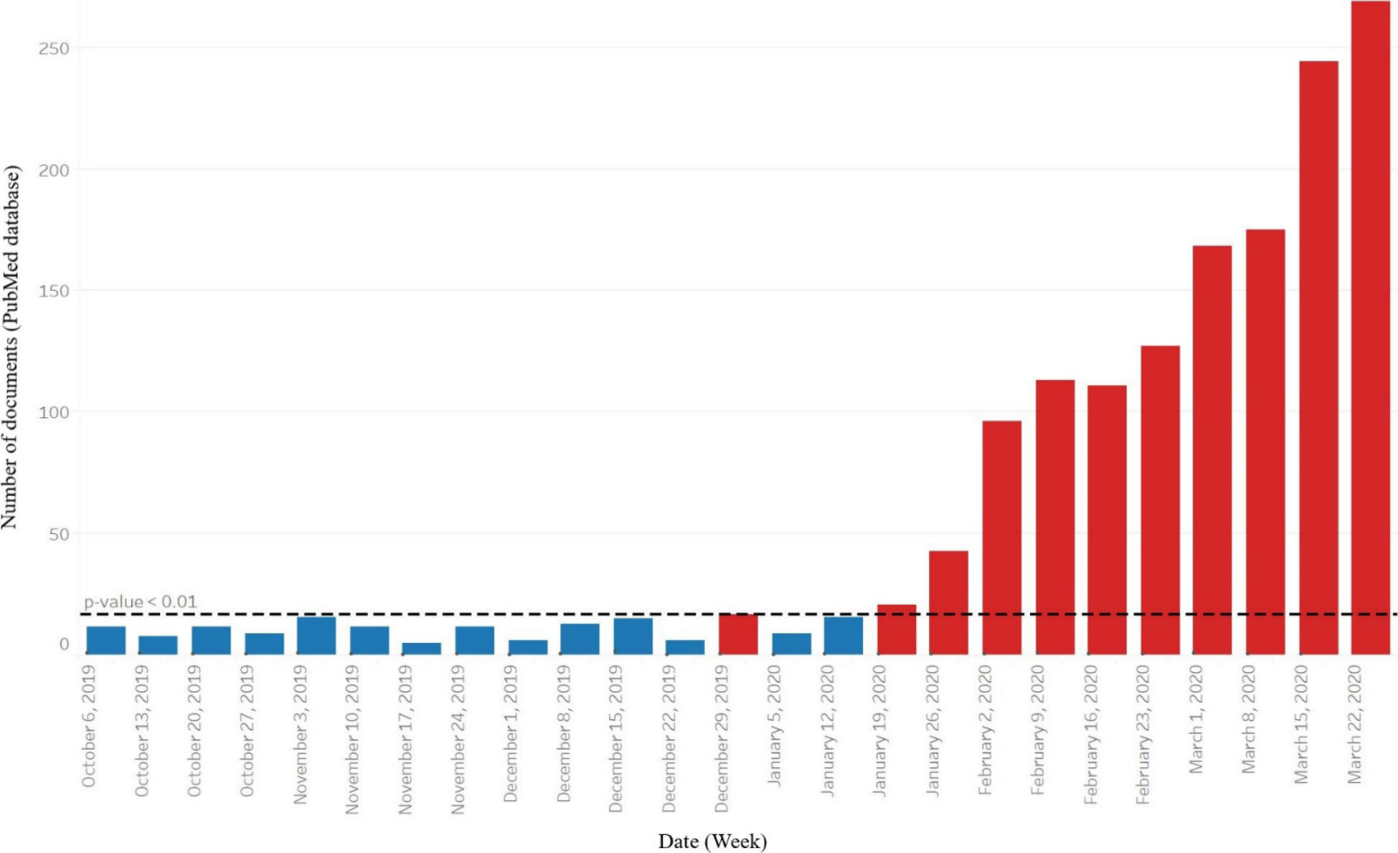
The Number of PubMed Publications Containing the Term Coronavirus in Their “Title/Abstract” From 6th October to 29th March. The term “Coronavirus” was searched in the articles published in the PubMed database. The figure shows the number of publications based on their Date Created (DA) and consists of articles published from 6th October to 29th March. As the figure shows, the sustained rise in the number of research papers occurred on 19-25th January, 3 weeks after WHO was notified (31st December), and 6 weeks after the epidemic started (8th December 2019). Dashed-line refers to the Number 16.68 in which a 99% significant increase occurs, and all of the numbers higher than it are statistically significant (red bars). Each date shows the first day of the week

The mean (SD) number of articles per week for the control period (from 6th January to 8th December) was 9.8 (2.9), and this level was considered as the baseline. After the epidemic start-time on 8th December 2019 [1, 16], the first week that showed a significant increase in the number of articles than the baseline was 19-25th December (documents number =21, *p*< 0.001) and, such increase was repeated in the next weeks (Fig. 1). Notably, 29th December to 4th January also showed a significant increase in the number of articles (documents number= 17, *p*< 0.01); however, this result was not repeated in the next 2 weeks, and we did not consider this record as a significant increase. Therefore, the time range between rising in the number of articles and the epidemic start-time was 6 weeks and, the time range between this rising and notifying to WHO of the epidemic (31st December 2019 [22]) was 3 weeks.

**Fig. 2:**
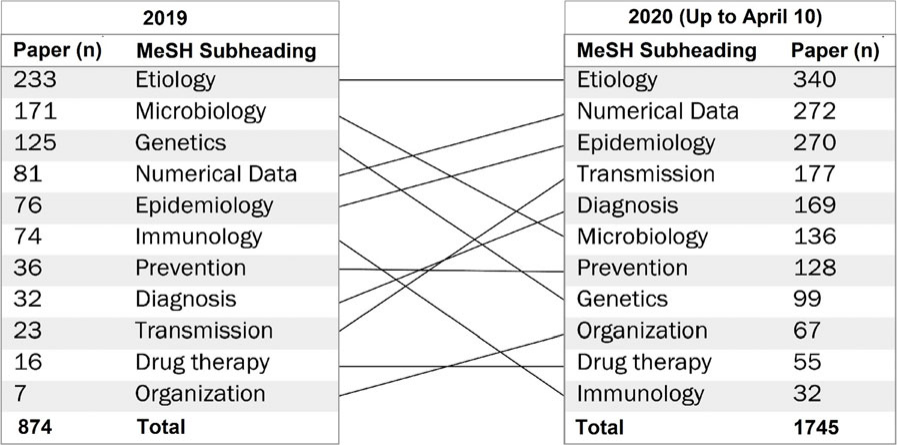
The most frequent MeSHs assigned to the coronavirus-related articles before and during the COVID-19 epidemic. Desired MeSH subheadings were searched in the articles that have the term “coronavirus” in their “title/abstract” and indexed in the year 2019 and 2020 (up to 10th April). MeSH terms were used as the indicator of the articles’ topics. The left and right columns show the number of records, and the lines connect the same subheadings in these 2 years. In the figure, the terms “organization,” “numerical data,” and “prevention” stand for organization & administration, statistics & numerical data, and prevention & control, respectively

The number of search results for before and during the COVID-19 epidemic was 874 and 1745, respectively. The three most frequent MeSH subheadings assigned to the articles about coronaviruses indexed in 2020, up to 10th April, were etiology (340 articles), statistics & numerical data (272 articles), and epidemiology (270 articles) (Fig. 2). In contrast, the three most common MeSH subheadings for articles of the year 2019 were etiology (233 articles), microbiology (171 articles), and genetics (125 articles). Statistics & numerical data, epidemiology, transmission, diagnosis, and organization & administration were five topics that their ranks in frequency were increased in 2020 than in 2019. Besides, the increase in relative frequency was significant for all these topics, along with prevention and control (0.041 vs. 0.071, *p*-value < 0.001).

### The SARS epidemic

**Fig. 3:**
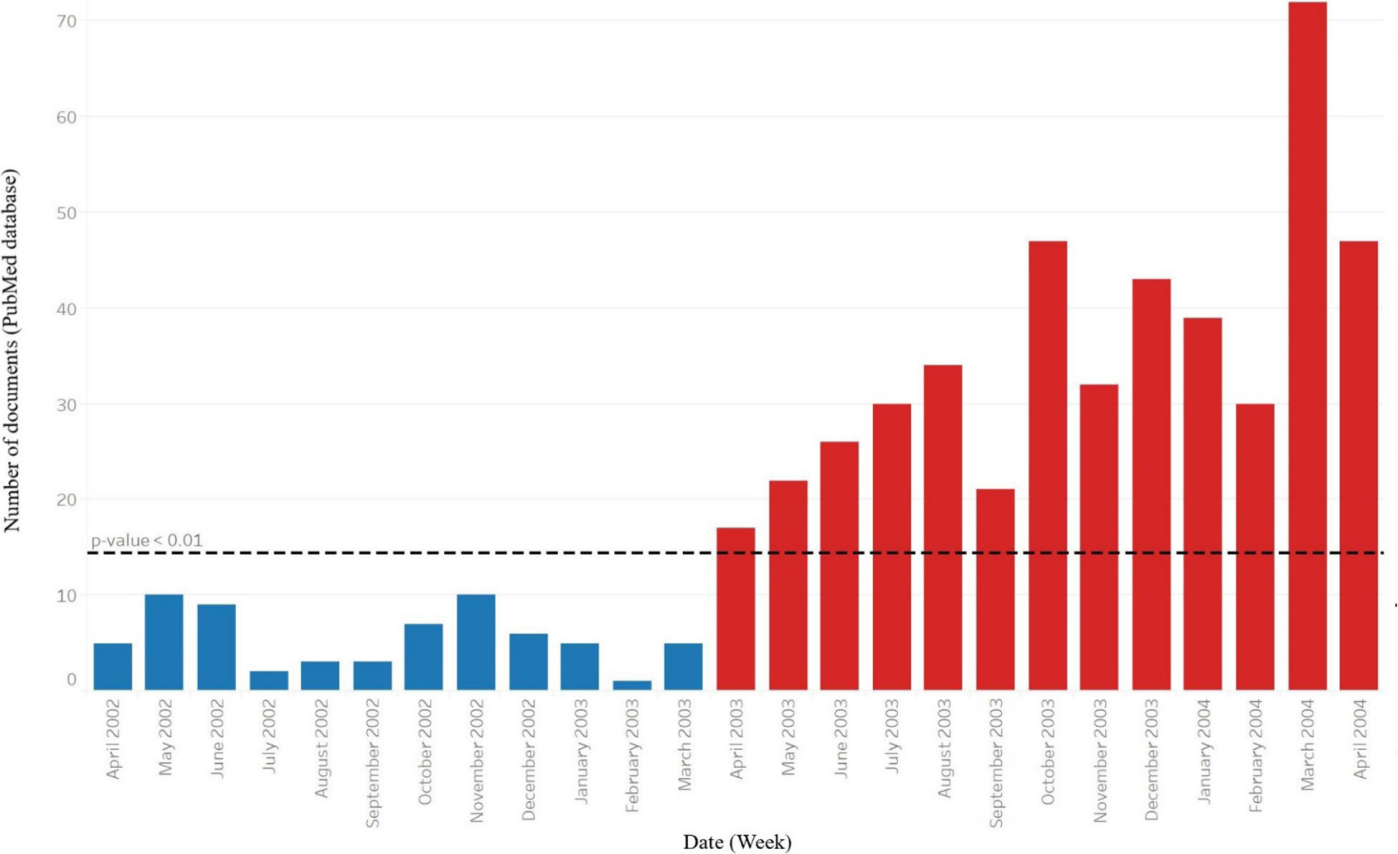
The Number of PubMed Publications Containing the Term Coronavirus in Their “Title/Abstract” From April 2002 to April 2004. The Term Coronavirus was searched in the “title/abstract” of articles published in the PubMed database. The figure shows the number of publications based on their Date Created (DA) and consists of articles published from April 2002 to April 2004. The first Cases of the SARS epidemic occurred in November 2002, but unfortunately, china reported the SARS epidemic officially on 11 February 2003. The rise in the number of publications occurred approximately two months later, in April 2003. Dashed-line refers to the Number 14.39 in which a 99% significant increase occurs, and all of the Numbers higher than it are statistically significant (red bars)

The mean (SD) number of articles per month added to the PubMed database in the control period (1st November 1998 to 1st November 2002) was 6.7 (3.27). We considered this number of articles per month as the baseline. In the epidemic course, which started in November 2002 [17], the first month, which showed a significant increase in the number of articles compared to the baseline, was April 2003 (documents number= 17, *p*< 0.001) (Fig. 3). This significant increase than the baseline was also repeated in the next few months. Therefore, the rise in the number of indexed articles occurred approximately 5 months after the epidemic started, and 2 months after the epidemic was reported by WHO (February 2003 [8]).

**Fig. 4:**
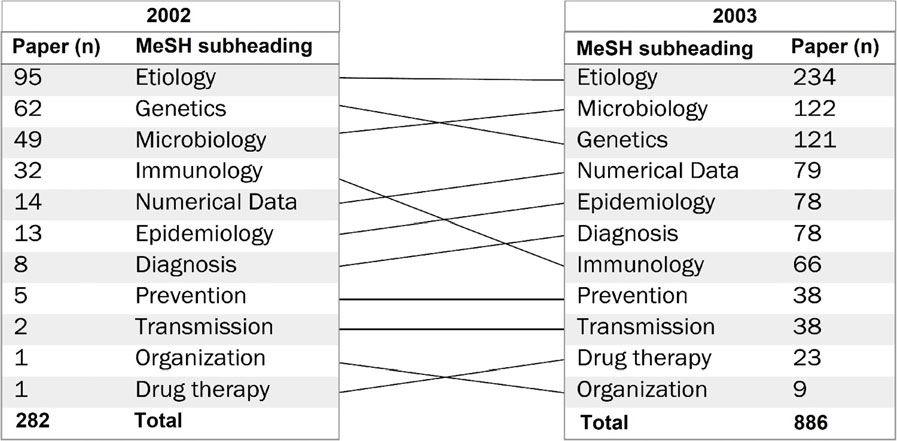
The most frequent MeSHs assigned coronavirus-related articles before and during the SARS epidemic. The term “coronavirus” and each shown subheadings were searched in the “title/abstract” of articles indexed in 2002 and 2003. The left and right columns show the number of records, and the lines connect the same subheadings in these two years. The terms “organization,” “numerical data,” and “prevention” stand for organization & administration, statistics & numerical data, and prevention & control, respectively

The number of papers included in the study for before and during the SARS epidemic was 282 and 886, respectively. The three out of eleven most common MeSH terms assigned to the articles that are about coronaviruses and indexed in 2003 were as follow etiology (234 articles), microbiology (122 articles), and genetics (121 articles) (Fig. 4). These three MeSH subheadings were also the most frequent ones in 2002, and the frequency was 95 articles for etiology, 62 articles for genetics, and 49 articles for microbiology. The rank of five topics in frequency was increased in 2003 than 2002 that comprises: microbiology, statistics & numerical data, epidemiology, diagnosis, and drug therapy. However, diagnosis and transmission were the topics that their relative frequency was increased significantly (0.028 vs. 0.088 for diagnosis, 0.007 vs. 0.042 for transmission, *p*-value < 0.01).

### The Ebola epidemic

**Fig. 5:**
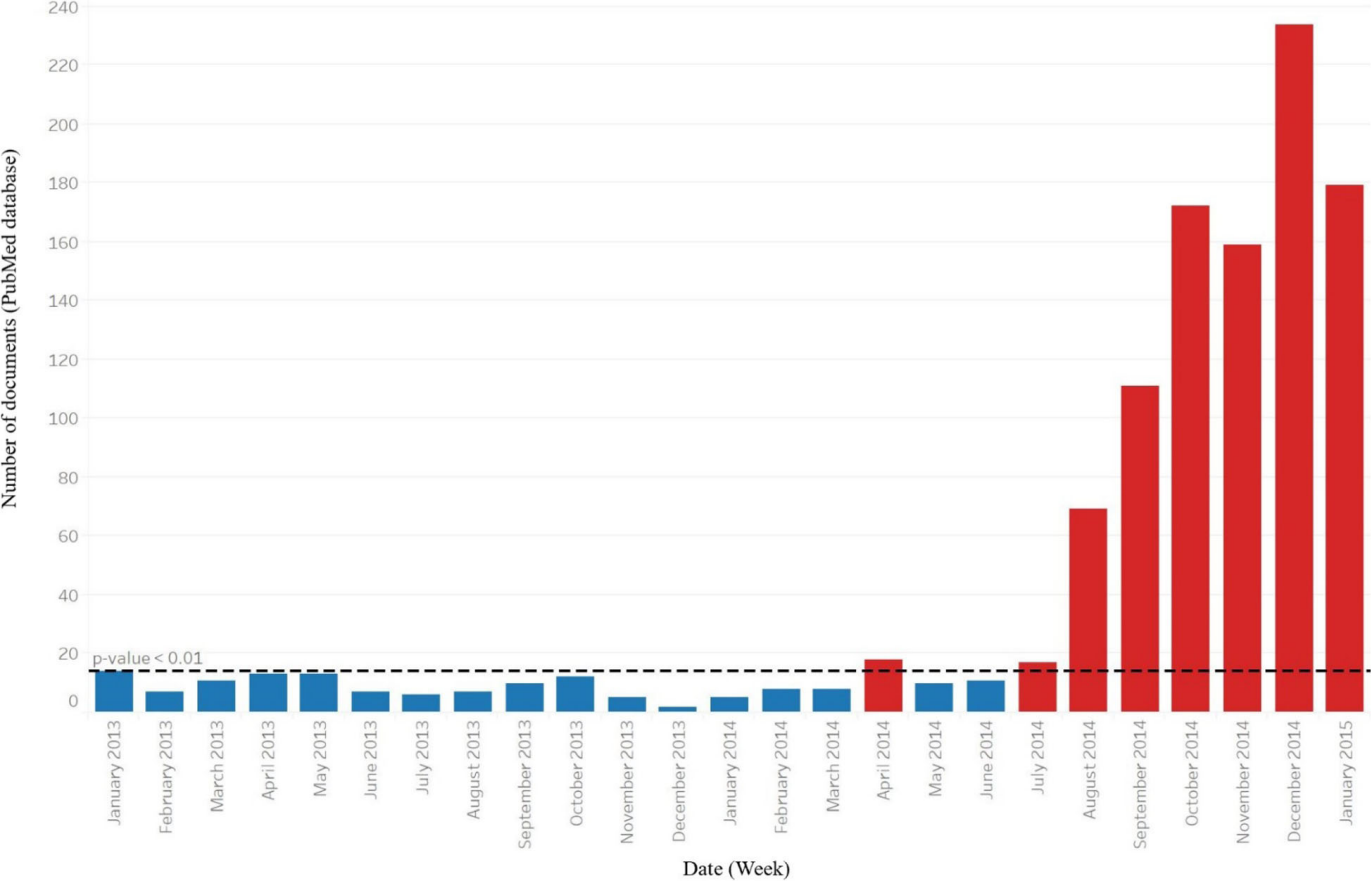
The Number of PubMed Publications Containing the Term Ebola in Their “Title/Abstract” From January 2013 to January 2015. The Term Ebola was searched in the “title/abstract” of articles published in the PubMed database. The figure shows the number of publications based on their Date Created (DA) and consists of articles published from January 2013 to January 2015. The first Cases of Ebola epidemic infected in December 2013, and the epidemic was reported on 23rd March 2014. The figure shows that the Rising in the number of publications occurred four months later, July 2014. Dashed-line refers to the Number 14.25 in which a 99% significant increase occurs, and all of the Numbers higher than it are statistically significant

The mean number of articles per month indexed in the control period (1st December 2009 to 1st December 2013) was 8.1 (2.62), and this level was considered as the baseline. In the epidemic course, a sustained significant increase in the documents number compared to the baseline was started from July 2014 (documents number=17, *p*< 0.001) (Fig. 5). April 2014 also showed a significant increase in the documents number than the baseline (documents number=18, *p*< 0.001); nevertheless, this increase was not repeated in the next 2 months. This epidemic started in West Africa in December 2013 and was declared in March 2014 by WHO [18, 23, 24]; therefore, the sustained rise in the number of indexed articles occurred 7 months after the epidemic start-time and 4 months after the epidemic declare-time.

**Fig. 6:**
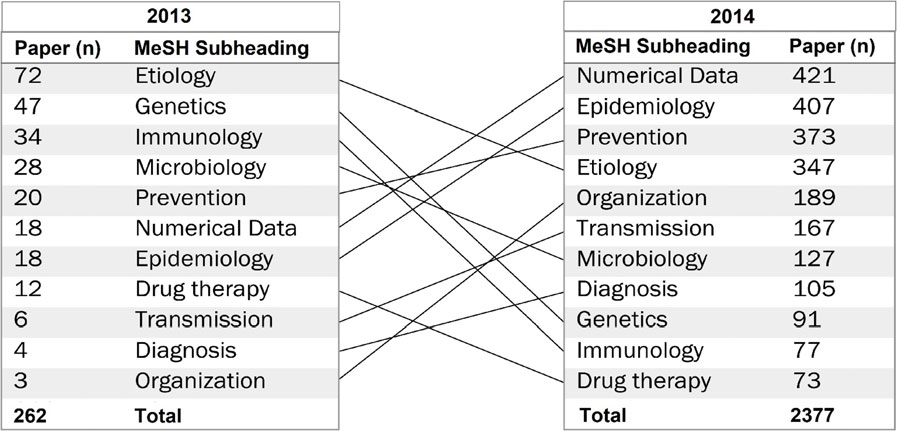
The most frequent MeSHs assigned to the Ebola-related articles before and during the Ebola epidemic start-time. We searched each subheading shown in the figure as the MeSH subheading and the term “Ebola” in the “title/abstract” of the articles indexed in 2013 and 2014. The figure shows the number of records, and the lines connect the same subheadings in these two years. The terms “organization,” “numerical data,” and “prevention” stand for organization & administration, statistics & numerical data, and prevention & control, respectively

The number of papers included in the study for before and during the Ebola epidemic was 262 and 2377, respectively. The three most frequent MeSH subheadings assigned to the articles that are about the Ebola virus disease and added to the PubMed database in 2014 were statistics and numerical data (421 articles), epidemiology (407 articles), and prevention and control (373 articles) (Fig. 6). However, in 2013, the most common MeSH subheadings in the articles that are about the Ebola virus disease were etiology (72 articles), genetics (47 articles), and immunology (34 articles). The six topics that their ranks in frequency were increased comprises statistics & numerical data, epidemiology, prevention & control, organization & administration, transmission, and diagnosis. Similarly, the increase in relative frequency was significant for these topics except diagnosis (0.015 vs. 0.044, *p*-value= 0.012).

## Discussion

This study shows that the speed of sharing research results was improved in the COVID-19 epidemic than the SARS and Ebola epidemics. This improvement happened due to the reduction in two time-lags in the case of the COVID-19 epidemic in comparison with the SARS and Ebola epidemics. First, the time lag between the COVID-19 epidemic start-time and declare-time (lag of declaring), second, the time lag between the epidemic declare-time and occurring a significant increase in the number of articles (lag of sharing). The lag of declaring for the COVID-19 epidemic was approximately 3 weeks, which was much shorter than this lag in the case of the SARS and Ebola epidemics (three months) [8, 22, 23]. Besides, the lag of sharing for the COVID-19 epidemic was three weeks (Fig. 1). In comparison, the lag of sharing was 2 months for the SARS, and 4 months for the Ebola epidemic (Figs. 3, 5).

Reduction in the lag of declaring led to a more rapid awareness of the outbreak and reacting to it. Although china’s government transparency was not ideal at the start of the COVID-19 outbreak [25], the first official reports of the epidemic were released sooner compared to the experience of the SARS outbreak [8]. Similarly, the Ebola epidemic was not reported for 3 months, which facilitated several transmission chains to progress [9].

The marked decrease in the lag of sharing research results after the COVID-19 epidemic declaration could be a consequence of enhancement in the data-sharing mechanism [26]. Changing global norms for sharing data and results in global health emergencies was reached after sensing shortcomings in data-sharing mechanisms through the Ebola virus disease outbreak in West Africa [5, 10]. This change led to the agreement of open, timely, and transparent sharing of data and results for all kinds of articles in public health emergencies such as the COVID-19 epidemic [11]. However, there is a constructive debate among research society on the rules and ethics regarding the data sharing in epidemics. Once the research community considers this kind of data sharing desirable to provide real-time guidance for epidemiologists, politicians, and modelers [27, 28], some highlights the importance of ensuring openness and proper attribution to prevent unethical issues and research misconducts [26]. Besides, sharing pre-publication datasets, as happened in the COVID-19 epidemic [29], could arise cautions about lacking extensive quality control before the data release. However, some believe that these cautions come with more significant benefits of early data release [30]. All these controversies could lead to the further evolution of the data-sharing mechanism in public health emergencies.

The study also showed a difference in the most frequent topics investigated in these three epidemics and that some topics were investigated more frequently after the epidemics started. The evidence is that Etiology, statistics & numerical data, and epidemiology were the three most frequent topics investigated in the COVID-19 epidemic (Fig. 2). However, Etiology, microbiology, and genetics in the SARS epidemic, and statistics & numerical data, epidemiology, and prevention in the Ebola epidemic were more frequently studied than other topics (Figs. 4, 6). Statistics & numerical data, epidemiology, and diagnosis were three topics that their ranks in frequency were increased after the three epidemics started (Figs. 2, 4, and 6). However, transmission was the topic that its relative frequency was increased significantly after the three epidemics started. These results represent such topic’s importance in the epidemics spread control. By contrast, immunology and genetics were two topics that their ranks in frequency were decreased after every three epidemics started.

To our knowledge, this is the first study that analyses research dynamics in the COVID-19 epidemic in comparison with other epidemics to show both improvements and shortcomings in data sharing against public health emergencies. However, we had some limitations in this study; some are mentioned as follows. First, we focused on published peer-reviewed papers indexed in the PubMed database because it is the most comprehensive bibliographic database in biomedicine and life science and enables easy access to the Create Date (CRDT) of each article [19]. Although, article indexing in PubMed takes some time, and this delay might confound this study’s results. Searching preprint services instead of PubMed is another choice that has its own limitations because Preprints are preliminary reports that have not undergone peer review and have no place in informing clinical practice and policy-makers. Another limitation was finding a feasible and valid method to determine article topics. Here, MeSH terms assigned to each article were used as the articles topic indicator rather than the manual screening of the articles’ title/abstract. However, Mesh term assignment to documents is manual too, and it is done by biomedical subject specialists based on the context of the whole document and not only the abstract and the title. Thus, they contain some information that may not be inferred from the title or the abstract of the article; therefore, it may not be the document’s main topic [31].

## Conclusions

The results indicate that the speed of sharing results in the COVID-19 epidemic was much higher than the SARS and Ebola epidemics and that there was a difference in the most frequent articles’ topics investigated in these three epidemics. The study highlights the necessity of taking preventive and preemptive measures to reduce the lag of declaring epidemics and lag of sharing research results in the next public health emergencies. Considering that disastrous epidemics could be initially evaluated as “common infectious diseases,” countries should be committed to being more sensitive and transparent in reporting epidemics. Furthermore, Incentives for sharing data should be created and tailored along with proposing more quality control processes before public dissemination of preliminary results. The study also suggests that urgent research topics that are needed to be investigated to encounter health emergencies should be determined, focused, and financially supported in the epidemic periods.

## Data Availability

The datasets used and/or analyzed during the current study are available from the corresponding author on reasonable request.

## Abbreviations

COVID-19: Coranavirus Disease 2019
CRDT: Date-Create
MeSH: Medical Subheading
